# No guts, no glory: IL-26 in host-microbiota interactions

**DOI:** 10.1038/s44318-025-00589-9

**Published:** 2025-10-22

**Authors:** Kathryn Wright, Serge Mostowy

**Affiliations:** Department of Infection Biology, London School of Hygiene and Tropical Medicine, London, UK

**Keywords:** Immunology, Microbiology, Virology & Host Pathogen Interaction

## Abstract

A recent study shows that innate lymphoid cells produce the bactericidal cytokine interleukin-26 to control microbiota composition and gut epithelial homoeostasis.

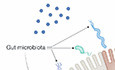

## Teaser

The gastrointestinal tract is composed of epithelial cells, immune cells and microbiota that interact dynamically to preserve homoeostasis. In this issue of The EMBO Journal, Salloum et al, (2025) exploit zebrafish models to show that bactericidal activity of interleukin-26 (IL-26), a poorly understood cytokine produced by innate lymphoid cells (ILCs), controls microbiota composition and gut epithelial homoeostasis.

The gut is a highly organised system of specialised epithelial cells (enterocytes) and immune cells that interact with resident microbiota to support human health. The interplay of immune cells with microbiota in the regulation of gut epithelial homoeostasis is the subject of intense investigation, particularly during early development. Identifying factors that regulate microbiota composition during early development is crucial in understanding how microbiota contribute to lifelong health.

A key player in maintenance of intestinal health is interleukin-26 (IL-26). While IL-26 has been associated with a range of inflammatory conditions affecting the gut, including inflammatory bowel disease (IBD) (Bos**á**ková et al, 2024), the in vivo function is mostly unknown, primarily due to the absence of IL-26 in mice. Considering that zebrafish possess a single orthologue of human IL-26 and its receptor chains IL10RB and IL20RA, zebrafish larvae provide an ideal model to study IL-26 receptor-dependent and -independent regulation of microbiota and epithelial homoeostasis in early development.

Zebrafish are an established non-mammalian vertebrate model to study gut biology and infection dynamics (Flores et al, 2020). Taking into account their biological properties (high fecundity, optical accessibility, and genetic tractability), zebrafish are exceedingly suited for the application of innovative technologies, including advanced microscopy techniques and single-cell RNA-sequencing (scRNA-seq) to investigate complex biological questions. Work using zebrafish larvae (where adaptive immunity is functional from 5 weeks post fertilisation) has primarily focused on innate components, such as macrophages and neutrophils (Gomes and Mostowy, 2020), however the full breadth of larval immune components is only beginning to emerge (Andersen-Civil et al, 2025).

Innate lymphoid cells (ILCs) play a major role in bridging innate and adaptive immune systems. They are tissue resident lymphocytes which lack traditional antigen receptors yet possess innate immune cell function and are able to rapidly produce cytokines and trigger immune signalling. ILCs predominantly reside within the gut, and their role in barrier homoeostasis and immune crosstalk is well established (Yoo and Oh, 2023). In previous work, Hernandez and colleagues used scRNA-seq to generate an atlas of zebrafish lymphocytes during tissue homoeostasis and after immune challenge and discovered that diverse ILC subsets are present in the zebrafish gut (Hern**á**ndez et al, 2018). In this issue of The EMBO Journal, Salloum and colleagues significantly advance our understanding of gut immunity, demonstrating in vivo consequences of IL-26 from ILCs on microbiota composition and epithelial homoeostasis in early development (Salloum et al, 2025).

By exploiting zebrafish, the authors present a first animal model to study impact of IL-26 on gut homoeostasis in vivo. Using CRISPR-Cas9 to generate transgenic zebrafish in which IL-26 is deleted (*il26*^−/−^), the authors observed increased epithelial cell proliferation and DNA damage in the posterior early life gut in a receptor-independent manner. Previous work has shown that human IL-26 protein has receptor-independent functions, including direct lysis of bacteria through pore formation (Gilliet and Modlin, 2024). In the case of zebrafish IL-26 protein, the authors revealed that intrinsic bactericidal activity is conserved through in vitro antimicrobial assays, demonstrating killing of common gut microbes *Escherichia coli* and *Pseudomonas aeruginosa*. The conservation of intrinsic bactericidal activity was further examined through in vivo infection assays with the gut pathogen *Edwardsiella tarda* (Rendueles et al, 2012). Infection of larvae with *E. tarda* confirmed intrinsic bactericidal activity of IL-26, with *il26*^*−/*−^ larvae showing an increased bacterial load and infection-associated mortality.

The authors next hypothesised that a lack of IL-26 may influence microbiota composition to regulate gut epithelial homoeostasis. To test this, microbiota composition was profiled using 16S rRNA-seq on dissected guts from *il26*^−/−^ and wildtype larvae, discovering that IL-26 shapes intestinal microbiota composition in the early life. The loss of IL-26 skewed microbiota composition and impacted the proportion of clinically relevant *Enterobacteriaceae* species, underscoring the critical role of IL-26 in inflammatory gut conditions. To interrogate the role of microbiota in increased enterocyte proliferation and DNA damage, the authors performed bulk RNA-seq on dissected guts from *il26*^−/−^ and wildtype larvae reared under germ-free conditions; however, they observed no differences in cell cycle or DNA repair genes. Further, co-housing wildtype germ-free larvae with *il26*^−/−^ larvae under conventional conditions did not affect markers for cell proliferation or DNA damage, suggesting that IL-26 is most crucial during early development. Microbiota transfer from *il26*^-/-^ to wildtype did not increase epithelial cell proliferation and DNA damage in wildtype zebrafish, suggesting that microbial agents responsible for phenotypes in *il26*^−/−^ larvae are unable to colonise wildtype gut. Future work may identify bacterial species failing to colonise wildtype larvae, indicating a potential role for these species in inducing cell proliferation and DNA damage in enterocytes from *il26*^−/−^ larvae.

To identify the cellular source of IL-26 and clarify the mechanism through which this cytokine can mediate microbiota composition, the authors probed previously generated scRNA-seq datasets of larval zebrafish IBD models and identified ILCs as the primary *il26-*expressing cells, present in zebrafish as early as 5 days post fertilisation. IL-26 has been suggested to regulate gut homoeostasis through induction of proinflammatory immune pathways in the adult fish gut (Zeng et al, 2024), however, emergence of ILCs in the zebrafish larval gut and their cytokine production profile was previously unknown. Here, Salloum and colleagues highlight the importance of ILCs and the cytokines they produce in gut homoeostasis during microbial colonisation in early development (Salloum et al, 2025).

Despite gut dysbiosis observed in larval *il26*^−/−^ zebrafish, consequences of IL-26 loss on epithelial cell proliferation and DNA damage were not observed in juvenile zebrafish. This could be attributed to the maturation of the immune system at later developmental stages. Further profiling of microbiota in juvenile and adult *il26*^−/−^ zebrafish could clarify whether dysbiosis observed in larval stages is resolved (potentially through adaptive immunity) or whether it is mitigated by compensatory mechanisms (that limit its impact on gut epithelium). Progressing study of IL-26 in adult zebrafish would also provide an important model for inflammatory gut conditions and, as zebrafish immune complexity is further uncovered (Resseguier et al, 2023; Andersen-Civil et al, 2025), allow for study of ILC/IL-26-dependant signalling and cooperation with adaptive immunity.

**Figure 1:**
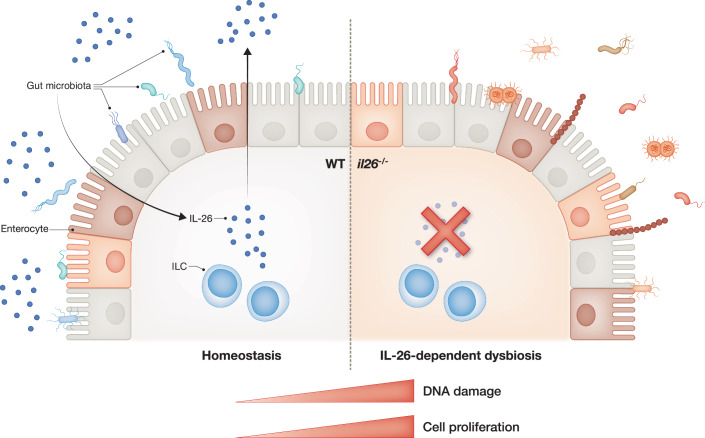
Working model. (Left) During early development, interleukin-26 (IL-26) expression is induced in innate lymphoid cells (ILCs) as microbes colonise the developing gut. IL26 works to maintain microbiota composition through bactericidal activity. This process ensures that gut epithelial cells (enterocytes) sustain a baseline level of DNA damage and proliferation for cellular turnover. (Right) The loss of IL-26 and its bactericidal activity (*il26*^−/−)^ alters microbiota composition to increase DNA damage and proliferation, resulting in gut dysbiosis.

In summary, these findings reveal key mechanisms by which host-microbiota interactions during early development, mediated by ILC-derived IL-26, protect against dysbiosis (Fig. 1). This study is a landmark example of utilising zebrafish models to address questions paramount to human health. It is exciting to consider these mechanisms and therapeutic implications, for example, the development of IL-26 supplementation for IBD patients with elevated levels of *Enterobacteriaceae*, microbiota transfer in genetically predisposed IBD patients, and the use of IL-26 to control gut bacterial infections.
